# Quantifying unmet prevention needs among MSM in Europe through a multi-site bio-behavioural survey

**DOI:** 10.2807/1560-7917.ES.2018.23.49.1800097

**Published:** 2018-12-06

**Authors:** Massimo Mirandola, Lorenzo Gios, Nigel Sherriff, Ulrich Marcus, Igor Toskin, Magdalena Rosinska, Susanne Schink, Sharon Kühlmann-Berenzon, Barbara Suligoi, Cinta Folch, Christiane Nöstlinger, Sonia Dias, Danica Stanekova, Irena Klavs, Saulius Caplinskas, Alexandru Rafila, Carolina Marin, Ivailo Alexiev, Lev Zohrabyan, Teymur Noori, Cinthia Menel-Lemos, on behalf of the SIALON II Network

**Affiliations:** 1 Infectious Diseases Section, Department of Diagnostics and Public Health, University of Verona, Verona, Italy; 2 School of Health Sciences, University of Brighton, Brighton, United Kingdom; 3 Department of Infectious Disease Epidemiology, Robert Koch Institute, Berlin, Germany; 4 Department of Reproductive Health and Research, World Health Organization, Geneva, Switzerland; 5 Department of Epidemiology of Infectious Diseases and Surveillance, National Institute of Public Health – National Institute of Hygiene, Warsaw, Poland; 6 Department of Public Health Analysis and Data Management, Public Health Agency of Sweden, Solna, Sweden; 7 Centro Operativo AIDS, Dipartimento di Malattie Infettive, Parassitarie ed Immunomediate, Istituto Superiore di Sanità, Rome, Italy; 8 Centre d’Estudis Epidemiològics sobre les Infeccions de Transmissió Sexual i Sida de Catalunya (CEEISCAT), Dept Salut, Generalitat de Catalunya / CIBER Epidemiologia y Salud Pública (CIBERESP), Barcelona, Spain; 9 Department of Public Health, Institute of Tropical Medicine, Antwerp, Belgium; 10 Faculty of Psychology, University of Vienna, Austria; 11 Escola Nacional de Saúde Pública, Centro de Investigação em Saúde Pública & GHTM, Universidade NOVA de Lisboa, Portugal; 12 NRC for HIV/AIDS, Slovak Medical University, Bratislava, Slovak Republic; 13 National Institute of Public Health, Ljubljana, Slovenia; 14 Centre for Communicable Diseases and AIDS, Mykolas Romeris University, Vilnius, Lithuania; 15 National Institute of Infectious Diseases Prof. Dr. Matei Bals, Bucharest, Romania; 16 ACCEPT, Bucharest, Romania; 17 National Reference Laboratory of HIV, National Center of Infectious and Parasitic Diseases, Sofia, Bulgaria; 18 Regional Support Team Joint United Nations Programme on HIV/AIDS (UNAIDS), Moscow, Russia; 19 European Centre for Disease Prevention and Control, Stockholm, Sweden; 20 Consumers, Health, Agriculture and Food Executive Agency (Chafea), Luxembourg; 21 Members of the the Sialon II Network have been acknowledged at the end of this article

**Keywords:** GAM indicators, MSM, HIV, bio-behavioural survey, HIV weighted prevalence, HIV testing

## Abstract

**Introduction:**

The HIV epidemic represents an important public health issue in Europe particularly among men who have sex with men (MSM). Global AIDS Monitoring indicators (GAM) have been widely and jointly promoted as a set of crucial standardised items to be adopted for monitoring and responding to the epidemic.

**Methods:**

The Sialon II study, implemented in 13 European cities (2013-14), was a complex multi-centre integrated bio-behavioural cross-sectional survey targeted at MSM, with a concomitant collection of behavioural and biological (oral fluid or blood specimens) data. Rigorous sampling approaches for hard-to-reach populations were used (time-location sampling and respondent-driven sampling) and GAM indicators were calculated; sampling frames were adapted to allow weighted estimates of GAM indicators.

**Results:**

4,901 MSM were enrolled. HIV prevalence estimates ranged from 2.4% in Stockholm to 18.0% in Bucharest. When exploring city-level correlations between GAM indicators, prevention campaigns significantly correlated with levels of condom use and level of HIV testing among MSM.

**Conclusion:**

The Sialon II project has made an important contribution to the monitoring and evaluation of the HIV epidemic across Europe, integrating the use of GAM indicators within a second generation HIV surveillance systems approach and in participatory collaboration with MSM communities. It influenced the harmonisation of European data collection procedures and indicators via GAM country reporting and contributed essential knowledge informing the development and implementation of strategic, evidence-based HIV prevention campaigns for MSM.

## Introduction

The HIV epidemic represents an important public health issue in Europe, particularly among men who have sex with men (MSM). For example, across the entire European Region, one new HIV diagnosis case in every four is attributable to MSM. Moreover, in 15 EU/EEA countries (Austria, Croatia, Cyprus, Czech Republic, Germany, Greece, Hungary, Ireland, Malta, Netherlands, Poland, Slovakia, Slovenia, Spain and the United Kingdom), MSM accounted for roughly 50% of all new HIV cases [1,2]. According to the European Centre for Disease Prevention and Control (ECDC) and the World Health Organization (WHO), the 2014 HIV prevalence among MSM aged 25 years or younger is 2.9%, while for MSM older than 25 years, it is estimated to be 7.7%. Thirty-four percent of HIV cases attributed to sex between men are usually diagnosed before the age of 30 [1,3].

While over the last few years there has been a general increase in new HIV infections among MSM in the EU/EEA, among young MSM the increase has been particularly noticeable [4]. Indeed, the number of MSM aged 20–24 years newly diagnosed with HIV almost doubled between 2004 and 2013, while in MSM aged 30–39 years old there seems to be a relative stabilisation of new cases [1]. To reverse these trends, there is a need for strategic, large-scale comprehensive and complementary prevention measures such as increased HIV and Sexually Transmitted Infections (STIs) testing, condom promotion, access to pre-exposure prophylaxis (PrEP) and early treatment initiations [2].

The picture is arguably even more problematic in some Eastern European and Central Asian countries where, although the HIV epidemic among this population is often similar, the existence of relatively more stigmatising environment(s) are probably less conducive to the reporting of data that could potentially deepen the understanding of mechanisms of HIV transmission among MSM [5].

In order to target prevention strategies effectively and to monitor their impact at a local/regional and country level, a better understanding of the epidemiological patterns and identification of the most affected sub-populations, are key enabling factors in tackling the multifaceted HIV epidemic. In Europe this particularly relates to MSM and there is a clear need for a harmonised collection of reliable and comparable data on epidemiology and coverage of prevention measures in this population.

Consequently, international agencies (namely, the Joint United Nations Programme on HIV/AIDS (UNAIDS), WHO and ECDC) have called for countries to use robust surveillance and monitoring systems that adopt common and standardised indicators [6]. A key part of this international effort for harmonisation is the promotion and implementation of second generation HIV surveillance systems (SGSS), which collect and link biological and behavioural data [6]. The Global AIDS Monitoring indicators (GAM) are part of the SGSS and comprise a set of standardised items widely and jointly promoted by the WHO and UNAIDS [7,8].

However, despite concerted efforts by these international agencies, the implementation of such a methodological approach is patchy and requires both adoption and strengthening across countries [8]. Although country reporting to the UNAIDS GAM has improved consistently over the years, to date there remains considerable variability in response rates [8].

A recent review of the GAM reporting process has highlighted a lack of data relating to key populations. For example, in 2012, GAM data on key populations were reported in approximately only 30% of cases [9].

A standardised set of GAM indicators is crucial given their role in providing specific data and information to monitor the implementation of the Sustainable Development Goals (SDG) and the UNAIDS 90–90–90 strategy, recently endorsed by the European Commission (EC) Communication on ‘Next steps for a sustainable European future’ [10]. The EC has co-funded several projects in the area of HIV/AIDS, two of which have aimed specifically to implement a joint survey across different EU/EEA countries adopting the main principles of SGSS and the GAM approach as cornerstones. The two projects are the Sialon project [11] and the more recent Sialon II project [12,13]. In particular, in the Sialon II project a total of 13 countries with very different cultural and social environments with 30 institutions including public health institutions and non-governmental organisations (NGOs) were involved. The meaningful participation of Lesbian-Gay-Bisexual-Trans-plus (LGBT +) communities in all participating countries has been key in designing and implementing the study.

The value of the Sialon II project lays in the sampling approach (Time-Location Sampling (TLS) and Respondent-Driven Sampling (RDS)) and the use of GAM indicators. The methodology adopted allowed the weighted estimation of GAM indicators, presented in this manuscript. To our knowledge, this is the first paper delivering weighted estimates for GAM indicators in a large number of European cities set within the framework of a SGSS specifically targeting MSM.

The objectives of this paper are to present weighted estimates of the GAM indicators among MSM based on the Sialon II bio-behavioural survey implemented in 13 European cities (2013-14) and to discuss the usefulness of these GAM indicators in monitoring the HIV epidemic and responses across EU countries.

## Methods

Detailed descriptions of the study procedures and methods have been published elsewhere [12]. Here we present a short overview of the main methodological aspects.

### Study design

The Sialon II study was a complex multi-centre integrated bio-behavioural cross-sectional survey with a concomitant collection of behavioural data and biological data (oral fluid or blood specimens).

### Setting

The survey was implemented in 13 European cities. The decision to use TLS or RDS in each study site was based on preliminary formative research and organisational issues. TLS was adopted in nine cities: Brussels (Belgium), Sofia (Bulgaria), Hamburg (Germany), Warsaw (Poland), Lisbon (Portugal), Ljubljana (Slovenia), Barcelona (Spain), Stockholm (Sweden) and Brighton (United Kingdom (UK)). The setting for data collection included social and/or commercial venues and cruising settings preliminarily identified through formative research [14] and then selected randomly for data collection sampling calendars. RDS was used in four cities: Verona (Italy), Vilnius (Lithuania), Bucharest (Romania) and Bratislava (Slovakia). Regarding the latter, enrolment was based on the individuals’ social network and for the data collection locally accredited healthcare facilities (e.g. a hospital) were used. Data collection for all sites took place from April 2013 to November 2014.

### Sample size

The sample size estimation was carried out based on the results from the former Sialon I project and other available studies [11]. Based on assumptions of HIV prevalence in the target population of at most 15%, a precision of 5%, a significance level of 95% and a design effect of 2.0 provided a random clustered sample size calculation of 392 MSM per study site. Taking into account the possibility of invalid samples, a final target of 408 MSM per city for TLS and 400 for RDS was planned.

### Participants

Inclusion criteria were having had any kind of sex with another man during the previous year before enrolment, providing informed consent and agreeing to donate either an oral fluid (TLS) or blood specimen (RDS).

The exclusion criteria were being younger than the legal age of consent (18 years old) or having already participated in the study.

### Data sources/measurement

#### Behavioural data

Behavioural data were collected through a pen-and-paper self-administered questionnaire. Core items were developed in line with the GAM indicators [7]. To allow for sampling weight calculations, additional items were included in the questionnaires on the venues attendance (TLS) or on network size (RDS).

#### Biological data

Biological specimens were obtained from participants of both study arms (TLS/RDS). In cities where TLS was used, specimens were tested for HIV antibodies using Genscreen HIV 1/2 version 2, Bio-Rad (Marne la Roquette, France). A total IgG antibodies ELISA test Human IgG ELISA Kit 1x96, Quantitative/Immunology Consultants Laboratory was also used for oral fluid (OF) sample testing suitability and quality control. All HIV-reactive samples were re-tested with Vironostika HIV Ag/Ab, Biomerieux (Marcy-l'Étoile, France). Samples reactive to the first ELISA HIV test, but negative to the second, were classified as negative.

In cities where RDS was used, blood specimens were collected and processed for serum extraction according to the respective national guidelines for safety and quality assurance. Serum samples were tested with a HIV fourth generation ELISA/CLIA screening test. A Western Blot test was used to confirm the positive cases.

#### Variables

The variables used for the present analysis included the GAM indicators suggested for MSM target population [15]. All proposed items included in the GAM guidelines for MSM were used in the survey questionnaire. Numerators and denominators were defined as seen in Table 1.

**Table 1:** Definitions for numerators and denominators

GAM	Nominator	Denominator
1.11 (Prevention programme)	Number of MSM who replied ‘yes’ to both questions related to the prevention programmes as per GAM guidelines (knowledge of HIV testing services and condoms received in the last 12 months in the context of broad prevention campaigns – outreach service)	Total number of MSM who participated in the survey
1.12 (Condom use)	Number of MSM who reported that a condom was used the last time they had anal sex	Number of MSM who reported having had anal sex with a male partner in the last 6 months
13 (HIV testing)	Number of MSM who reported having been tested for HIV during the last 12 months and who knew their results	Number of MSM included in the sample
1.14 (HIV prevalence)	Number of MSM with a reactive HIV test (based on laboratory results)	Number of MSM tested for HIV in the context of the survey

GAM: Global AIDS Monitoring indicators; MSM: men who have sex with men.

### Statistical methods

The analysis was carried out according to the GAM indicator guidelines [15]. For all indicators, estimates were carried out with the following age disaggregation: < 25 years old and ≥ 25 years old. Age was calculated on the basis of the self-reported year of birth. Analyses were conducted using STATA Version 14.1 (StataCorp, College Station, Texas, United States). To allow calculation of the sampling weights, a specific procedure was devised on the basis of previous publications and methodological guidelines [12,16,17].

For the TLS survey, individual weights were assigned as the inverse of the product of the following: (i) the probability of the participant being at the sampled venue given he was at the sampled venue type (number of visits to sampled venue/number of visits to all types of venues); (ii) the length of the sampling time (out of all Venue-Day-Time units on the particular day) and (iii) the proportion of sampled individuals during the event in relation to the estimated number of visitors during the sampling event, a modification of the method proposed by Karon and Wejnert [17].

For the RDS survey, and in line with a RDS approach [18], weighted estimates were calculated using RDS Analyst (www.hpmrg.org), a suite of R commands developed by Handcock and colleagues (2015 RDS Analyst: Software for the Analysis of Respondent-Driven, Sampling Data, Version 0.52). Gile's Sequential Sampler approach was used for calculating the sampling weights. This approach is based on the inclusion probabilities of members of the sample which are based on reported network sizes [16]. This method is recommended when the sample is a significant fraction of the target population. Therefore, in order to use this method, population size estimates were carried out for each city. The calculation was based on the total number of inhabitants for the city area and the expected percentage of MSM (according to the consensus among the project’s scientists and according to the scientific literature, given there is currently no comprehensive and precise agreement among experts on MSM population size estimations within the general population) [19,20]. All point estimates were reported with their respective sample size, 95% Confidence Intervals (CI), and estimated design effect.

### Ethics

All procedures adopted in the present study were in line with the 1964 Helsinki declaration and its amendments. Survey protocols were approved by the appropriate ethics committee in each participating city as well as by both the WHO Research Project Review Panel (RP2) and the WHO Research Ethics Review Committee (ERC). The name or any other identifier of the MSM enrolled in the study was not collected. All respondents were entitled to collect their test result at a nominated centre indicated to the participant during study enrolment. In case of a positive result, further testing, counselling, clinical follow up and ARV treatment were provided in line with the respective national guidelines.

## Results

A total of 4,901 MSM were enrolled across the 13 participating cities. In TLS study sites, 3,596 participants were enrolled, while in RDS sites a total of 1,305 participants were enrolled. Participants enrolled per enrolment method/city, age mean, Min-Max, age group, are shown in Table 2. A detailed description of the sample is available in the Sialon II project report [13].

**Table 2:** Enrolment method, mean age, age group, by city, European Union cities (n = 13)

City	Recruitment type	Mean age (range)	Age group in years (GAM disaggregation)		Total
			< 25	25 +	
Barcelona	TLS	37.2 (19–79)	42	360	402
Brighton	TLS	35.1 (18–74)	67	344	411
Brussels	TLS	34.9 (18–68)	50	341	391
Hamburg	TLS	38.0 (18–79)	39	368	407
Lisbon	TLS	37.9 (19–76)	35	373	408
Ljubljana	TLS	30.5 (18–73)	121	273	394
Sofia	TLS	29.6 (18–58)	115	296	411
Stockholm	TLS	31.7 (18–81)	77	289	366
Warsaw	TLS	28.8 (18–71)	92	314	406
Bratislava	RDS	30.3 (18–62)	118	282	400
Bucharest	RDS	30.8 (19–58)	47	134	181
Verona	RDS	31.9 (18–70)	104	293	397
Vilnius	RDS	30.7 (19–59)	83	239	322

GAM: Global AIDS Monitoring indicators; RDS: Respondent-Driven Sampling; TLS: Time-Location.

Cities presented with white background: TLS survey. Cities presented with light blue background: RDS survey.

### GAM 1.11 (Prevention programmes)

In eight of the nine cities where TLS was implemented, more than half of respondents answered positively to both questions (Table 3, GAM 1.11). The sole exception was Warsaw where 28.8% (95% CI: 23.3–34.3) of the participants had been reached by a prevention programme. Where RDS was implemented, less than half of the MSM answered yes to both the GAM questions, except for the older participants (≥ 25 years) in Bucharest (50.7%; 95% CI: 38.4–62.9) and young MSM (< 25 years) in Vilnius (58.3%; 95% CI: 43.4–72.3).

**Table 3:** GAM indicators (weighted estimates) among MSM, design effect and number of participants, by city and age group, European Union cities (n = 13)

City	Age group < 25				Age group 25 +				Total			
	Sample size	Point estimate	95% CI	Estimated design effect	Sample Size	Point estimate	95% CI	Estimated design effect	Sample size	Point estimate	95% CI	Estimated design effect
**GAM 1.11 (Prevention programmes)**												
Barcelona	40	59.0	39.2–78.8	1.7	352	74.9	70.7–79.2	0.9	392	72.7	68.4–77.0	0.9
Brighton	63	64.0	43.8–84.1	2.8	328	54.5	39.8–69.2	7.5	391	56.3	45.3–67.2	4.9
Brussels	47	49.1	18.0–80.2	4.6	324	64.4	55.1–73.7	3.2	371	62.1	51.9–72.3	4.2
Hamburg	37	84.3	69.8–98.7	1.5	357	81.0	76.6–85.4	1.2	394	81.4	77.6–85.1	1.0
Lisbon	35	79.9	63.9–95.9	1.4	364	64.4	51.2–77.6	7.2	399	66.0	52.7–79.3	8.2
Ljubljana	119	48.6	38.8–58.3	1.2	265	50.9	44.4–57.4	1.1	384	50.4	44.4–56.4	1.4
Sofia	112	84.7	79.7–89.7	0.6	291	89.7	81.9–97.6	5.1	403	88.4	82.9–94.1	3.2
Stockholm	61	72.7	51.6–93.7	3.5	245	78.0	70.1–85.9	2.3	306	76.6	66.8–86.3	4.2
Warsaw	91	25.5	13.0–38.0	1.9	311	30.2	22.5–37.9	2.3	402	28.8	23.3–34.3	1.5
Bratislava	115	19.2	10.0–27.5	2.0	276	24.0	17.0–29.5	2.1	391	22.6	17.0–26.8	1.9
Bucharest	46	27.4	12.6–42.4	1.4	122	50.7	38.4–62.9	2.0	170	45.9	36.1–55.7	1.7
Verona	99	27.7	15.5–39.5	2.2	275	39.1	30.6–47.7	2.7	375	35.9	28.9–43.0	2.5
Vilnius	82	58.3	43.4–72.3	2.2	236	30.1	22.0–35.6	1.6	318	37.5	30.1–42.8	1.7
**GAM 1.12 (Condom use)**												
Barcelona	36	84.6	70.5–92.7	1.05	267	65.7	56.5–73.9	2.26	303	68.7	59.1–77.0	2.95
Brighton	46	56.8	34.9–76.3	1.88	222	51.7	40.9–62.4	2.81	268	52.4	41.3–63.4	3.48
Brussels	39	83.7	67.3–92.8	0.95	253	57.6	44.5–69.8	4.51	292	60.7	47.7–72.3	4.97
Hamburg	29	74.9	44.0–91.9	2.90	252	49.1	38.8–59.6	2.80	281	52.5	42.6–62.1	2.84
Lisbon	30	53.1	28.5–76.3	2.65	294	72.0	65.2–77.9	1.45	324	69.6	64.5–74.3	0.95
Ljubljana	91	57.6	35.8–76.8	3.35	204	47.1	37.7–56.6	2.13	295	49.5	42.6–56.5	1.50
Sofia	114	80.0	71.7–86.2	0.85	278	59.3	54.5–64.0	0.72	392	64.7	60.6–68.5	0.70
Stockholm	38	62.6	44.2–78.0	1.71	160	56.9	43.9–69.0	2.47	198	58.4	47.0–69.0	2.61
Warsaw	67	51.4	37.3–65.3	1.70	215	57.0	47.9–65.7	1.70	282	55.4	48.2–62.4	1.50
Bratislava	88	41.7	27.2–53.1	1.96	202	46.7	38.5–54.8	1.74	290	45.2	38.1–51.4	1.67
Bucharest	31	34.6	11.8–56.9	1.84	85	62.3	47.8–76.8	2.00	116	56.6	43.7–69.5	2.06
Verona	87	55.7	39.5–71.1	2.62	235	63.9	55.2–73.9	2.70	324	61.6	53.9–70.1	2.70
Vilnius	67	59.6	45.2–74.3	1.77	177	55.3	46.0–65.5	2.02	244	56.6	48.9–65.0	1.91
**GAM 1.13 (HIV testing)**												
Barcelona	41	62.5	53.2–71.8	0.4	345	63.6	53.1–74.0	3.9	386	63.0	52.6–73.4	4.7
Brighton	63	36.1	22.7–49.6	1.2	313	54.6	43.9–65.3	3.3	376	47.3	43.3–51.3	0.6
Brussels	50	42.5	18.6–66.4	2.9	317	72.1	59.8–84.4	5.7	367	68.1	56.4–79.8	6.0
Hamburg	37	69.5	53.0–86.0	1.1	350	53.3	41.1–65.6	5.1	387	53.6	43.4–63.7	4.2
Lisbon	34	21.1	12.5–29.8	0.4	360	63.3	56.0–70.6	2.0	394	60.9	53.4–68.4	2.4
Ljubljana	117	40.1	23.5–56.6	3.4	256	47.4	41.9–53.0	0.8	373	46.4	40.5–52.3	1.4
Sofia^a^	NA	NA	NA	NA	NA	NA	NA	NA	NA	NA	NA	NA
Stockholm	62	52.1	39.0–65.2	1.1	229	57.9	47.1–68.6	2.6	291	56.3	45.6–66.9	3.5
Warsaw	91	37.6	27.2–48.1	1.0	301	60.6	48.6–72.6	4.5	392	54.0	45.7–62.2	2.8
Bratislava	112	29.0	19.1–39.0	1.9	228	41.0	31.8–48.8	2.2	340	37.2	30.4–43.0	1.9
Bucharest	47	39.0	21.8–56.2	1.6	122	44.8	32.5–57.1	2.0	171	43.3	33.1–53.5	1.9
Verona	99	50.2	36.3–64.0	2.3	233	47.3	36.1–57.1	3.1	335	47.7	38.6–56.1	3.2
Vilnius	81	38.3	24.8–50.5	1.8	192	39.1	29.0–47.6	2.1	273	38.8	31.2–45.0	1.7
**GAM 1.14 (HIV prevalence)**												
Barcelona	42	0.8	0.1–6.6	0.5	358	16.4	12.4–21.5	1.2	400	14.2	10.1–19.5	1.2
Brighton	65	2.9	0.5–15.5	1.5	337	20.7	15.7–26.8	1.4	402	17.6	13.8–22.3	0.9
Brussels	49	0.5	0.1–4.3	0.3	330	14.4	8.6–23.0	3.1	379	12.3	7.6–19.4	4.9
Hamburg	37	1.9	0.3–9.9	0.6	353	8.1	4.1–15.4	3.3	390	7.5	3.9–13.8	3.6
Lisbon	33	1.2	0.1–9.2	0.5	338	18.9	14.2–24.8	1.4	371	17.1	12.4–23.0	2.6
Ljubljana	107	1.4	0.2–11.1	1.1	240	5.3	2.2–12.2	2.6	347	4.4	2.1–8.9	1.8
Sofia	99	0.1	0.0–16.1	0.1	262	3.9	1.0–14.0	4.1	361	3.0	0.9–9.1	0.8
Stockholm	73	0.0	0.0–0.0	0.0	283	3.4	1.5–7.4	1.3	356	2.4	1.1–5.2	2.2
Warsaw	92	1.6	0.7–3.7	0.3	313	9.7	5.7–15.9	1.9	405	7.2	4.3–11.9	2.0
Bratislava	118	2.1	0.0–4.7	1.5	282	5.3	1.8–8.4	2.2	400	4.3	2.2–6.2	1.4
Bucharest	47	11.6	2.1–21.0	1.1	134	18.5	8.4–28.7	2.4	183	18.0	9.1–27.0	2.6
Verona	104	3.8	0.0–9.6	3.0	293	12.2	3.8–20.8	5.6	400	9.6	4.5–14.9	3.5
Vilnius	83	0.0	0.0–0.0	NA	239	4.6	0.4–9.2	3.3	322	3.4	0.0–6.9	3.6

GAM: Global AIDS Monitoring indicators; MSM: men who have sex with men; NA: not available.

^a^Results from Sofia for GAM 1.13 are missing due to incorrect translation.

Cities presented with white background: TLS survey. Cities presented a light blue background: RDS survey.

Estimated design effect: The design effect is a correction factor used to adjust required sample size for cluster sampling.

Reference items: GAM 1.11 (i) Do you know where you can go if you wish to receive an HIV test?, (ii) In the last twelve months, have you been given condoms?; GAM 1.13 (i) Have you been tested for HIV in the last 12 months?, (ii) If yes: I don’t want to know the results, but did you receive the results of that test?; GAM 1.12 and 1.14 did not have reference items.

The highest percentages of MSM reporting to be reached with an HIV prevention programme in the last 12 months were reported in Sofia and Hamburg (88.4%; 95% CI: 82.9–94.1 and 81.4%; 95% CI: 77.6–85.1 respectively). In Bratislava and Warsaw, the lowest proportions of MSM participating in the survey had been reached by an HIV prevention programme (22.6%; 95% CI: 17.0–26.8 and 28.8%; 95% CI: 23.3–34.3, respectively).

In all cities (except Brighton, Hamburg and Lisbon), older participants (≥ 25 years) had been reached more over the last 12 months than the younger participants (< 25 years), although differences between these age categories were small in most cities.

### GAM 1.12 (Condom use)

Condom use according to the GAM definition, ranged from 45.2% (95% CI: 38.1–51.4) in Bratislava to 69.6% in Lisbon (95% CI: 64.5–74.3) (Table 3). With the exception of a few sites (Bratislava, Bucharest, Lisbon, Verona and Warsaw) condom use was higher for the young MSM category (< 25 years). Among older MSM (≥ 25 years), estimates of condom use varied between 46.7% in Bratislava (95% CI: 38.5–54.8) to 72.0% in Lisbon (95% CI: 65.2–77.9). It should be noted that for some cities, the number of participants in the younger age group was low and therefore the precision of the estimates is reduced.

### GAM 1.13 (HIV testing)

The level of HIV testing as per GAM guidelines is reported in Table 3. The highest proportions of participants (total) reporting having received an HIV test within the last 12 months and who also knew the result of that test, were reported in Brussels (68.1%; 95% CI: 56.4–79.8) and Barcelona (63.0%; 95% CI: 52.6–73.4). In Lisbon only 21.1% (95% CI: 12.5–29.8) of younger men reported a HIV test with the collection of the test result in the last year, which represented the lowest levels in the study. The second lowest estimate after Lisbon was found in Bratislava with 29.0% (95% CI: 19.1–39.0) followed by Brighton 36.1% (95% CI: 22.7–49.6) and Bucharest 39.0 (95% CI: 21.8–56.2). In several cities there were significant differences between age groups with regard to receiving an HIV test within the last 12 months and knowing the results. Participants from Brussels, Lisbon, and Warsaw reported differences greater than 20% for the two age groups.

### GAM 1.14 (HIV prevalence)

HIV prevalence was calculated based on the oral fluid-based laboratory testing for TLS and from serum-based laboratory tests for RDS. HIV prevalence estimates varied by city with the lowest level reported in Stockholm (2.4%; 95% CI: 1.1–5.2) and the highest level in Bucharest (18.0%; 95% CI: 9.1–27.0) (Table 3). Five cities had an HIV prevalence between 10–20% (Brussels, Barcelona, Lisbon, Brighton, Bucharest), three cities between 5–10% (Hamburg, Warsaw, Verona), and five cities below 5% (Stockholm, Vilnius, Ljubljana, Bratislava and Sofia).

### City-level correlations between GAM indicators

When exploring city-level correlations between GAM indicators (Figure) a significant correlation was found between prevention programmes indicator (defined as condom availability and testing site knowledge) and HIV testing (correlation coefficient 0.52, p-value = 0.006). In addition, a correlation was found between preventive programmes indicator and condom use (correlation coefficient 0.45, p-value = 0.022). Data suggest that the higher the prevention programmes indicator the higher the level of condom use and testing among MSM, in both age groups.

**Figure:**
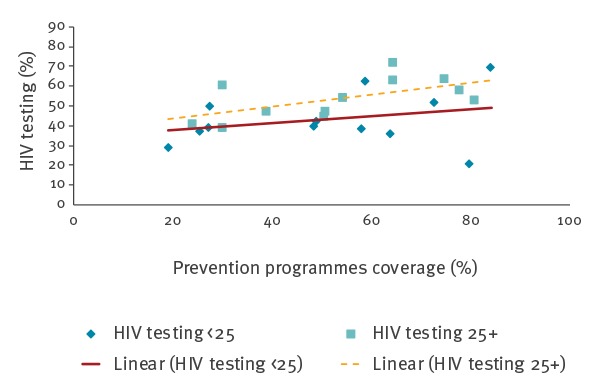
HIV testing (GAM 1.13) vs prevention programmes (GAM 1.11), European Union cities (n=13)

## Discussion

HIV remains a public health priority in the EU and the data from the Sialon II study has made an important contribution to the monitoring and evaluation of the HIV epidemic across Europe, by integrating the use of GAM indicators within a second generation HIV surveillance systems approach and in participatory collaboration with MSM communities. It has influenced the harmonisation of European data collection procedures and indicators via GAM country reporting and contributed essential knowledge informing the development and implementation of strategic, evidence-based HIV prevention campaigns for MSM.

The percentage of MSM reached with prevention programmes (as measured by GAM 1.11) showed significant differences both between and within cities. Although the use of different sampling methods can partially explain some of the differences between RDS vs TLS cities, the comparison among cities surveyed with the same sampling methodology provides valid indications on MSM prevention needs. In Warsaw, the number of individuals reached with prevention programmes is generally low, particularly for younger MSM. With the exception of Brighton, Hamburg, Lisbon and Vilnius, older MSM seem to be reached more frequently compared with younger MSM. Cities surveyed using RDS present, in general, a lower number of MSM reached with prevention programmes, and with the exception of Vilnius (where a specific programme was run by an NGO before and during the data collection period), younger MSM showed the lowest level. These estimates are consistent with the literature and suggest the need for targeted prevention programmes tailored to locations and communities that can also accommodate for the needs of sub-populations, such as young MSM, MSM who are tourists and bisexuals [21,22].

Data focusing on the GAM 1.12 (Condom use) indicate that in the majority of the surveyed cities, condom use with any kind of partner was lower for younger men. However, the sample sizes for younger men were small for several cities and the association between age and condom use was not consistent across all study cities. In another publication based on the same Sialon-II-dataset, authors looked at condom use from another perspective e.g. any anal intercourse without a condom during the previous 6 months and not just from the last sexual encounter. The authors reported that almost half of the HIV-uninfected individuals reported condomless anal intercourse (CLAI). This was reported slightly more often by men living in Central European study cities and more frequently with steady partners compared with non-steady partners [23].

Based on the estimates of this GAM indicator there is a clear need to either increase condom use among younger men or to complement prevention strategies by providing meaningful access to other similarly effective HIV prevention tools such as HIV pre-exposure prophylaxis. Despite the fact that condom and lubricant distribution is often considered a simple and somewhat naïve approach to facilitate condom use, it is commonly acknowledged in the literature that fear of disapproval and discrimination by healthcare providers can deter gay, bisexual and other MSM from accessing mainstream health services [18]. Indeed, this limitation, could reduce access to free condom distribution, as well as low threshold HIV and STIs testing in healthcare settings [24].

A study by Marcus et al. [25] modelling the relationship between unprotected anal intercourse (UAI) and HIV disclosure with the same study dataset, found that among those respondents being aware of being HIV positive, condom use with steady partners was higher than among HIV negative men. However, condom use with non-steady partners was also lower. Men unaware of being infected with HIV reported the lowest condom use with non-steady partners [23,25].

It is possible that a large number of MSM are unaware of their HIV status. It is estimated that in western and central Europe a noteworthy number of people at risk are not getting tested or can experience difficulties in being tested for HIV and STIs [2]. Based on surveillance data, ECDC estimates that a number of European countries may have a considerable proportion of late HIV diagnoses [2]. The testing behaviour as measured by the GAM 1.13 depicts a very different situation among the surveyed cities. In Barcelona, Hamburg, Sofia, Stockholm and Verona, approximately half of the participants (all ages) reported having received an HIV test within the last 12 months and knew the result of that test. In Brighton, Brussels and Warsaw more than half of older participants reported a known HIV test result within the last 12 months while among younger participants, it was below that level. In Lisbon only one in five younger men reported a known HIV test result in the last year which represented the lowest levels in the study.

In several cities there were differences between the two age groups with regards to receiving an HIV test within the last 12 months and knowing the results. For instance, participants from Brighton, Brussels, Lisbon, and Warsaw all reported differences of greater than 20% for the two age groups; however, considering the time span is limited to the last 12 months, the differences cannot be directly attributed to the age effect where, for example, older individuals may have had an increased opportunity to be tested over time. Furthermore, older participants were tested more frequently and within a shorter timeframe than the younger men, suggesting that increasing access to culturally sensitive HIV counselling, testing and antiretroviral therapy for MSM, found to have HIV, is an urgent health priority particularly for the younger generations. The current levels of HIV testing are insufficient to link gay, bisexual and other MSM with appropriate healthcare support shortly after acquiring HIV infection. Therefore, currently, testing frequencies can remain insufficient to effectively reduce the period of infectiousness of people who newly acquire HIV.

Alternative approaches such as the use of point of care tests (PoCTs) for HIV and STIs in low threshold community testing and LGBT venue-based testing, home collection testing, and HIV self-testing may represent effective approaches to increase diagnosis and linkage to care.

The problem of late diagnosis reflects a lack of access to and uptake of HIV testing and counselling services in many countries [26]. A late diagnosis also means that a person has remained unaware of their HIV status for an indeterminate length of time, thus increasing the risk of transmitting the virus. The most recent surveillance data showed that despite significant efforts dedicated to the prevention and control of HIV, the rate of new HIV diagnoses has not substantially declined in the EU/EEA, but it has increased substantially over the last decade in the European Region.

Although HIV prevalence as an epidemic indicator is not a good parameter of the HIV infection spread dynamic, it can be helpful to provide an indirect picture of the epidemic history and patterns for some cities. HIV prevalence estimates (GAM 1.14), as measured by testing biological specimens (and not self-reported serological status), highlight critical levels of HIV infections across Europe among MSM communities despite valuable and concerted public health efforts [15].

Brussels, Barcelona, Lisbon, Brighton and Bucharest when considered globally, present relatively high HIV prevalences within the range of 10–20%. That said, the HIV prevalence estimate for Bucharest is probably less reliable and interpretable compared with the other cities for two main reasons: the existence of an ostensible MSM sub-sample of injecting drug-users within the city sample, and the fact that the target number of MSM to be recruited was not reached (less than 50% of the estimated target sample was recruited). Compared to these relatively high HIV prevalences, more ‘intermediate’ levels of HIV prevalence were evident in four other participating cities (Hamburg, Warsaw, Verona, and Sofia) where it ranged from 5 to 10%. Finally, in the cities of Stockholm, Vilnius, Ljubljana, and Bratislava, relatively lower HIV prevalences were observed with results below 5%. Other smaller studies carried out in some of these cities (with different or similar sampling methods) have produced similar results [11,27].

When exploring city-level correlations between GAM indicators, the data confirmed that prevention programmes correlate with both the level of condom use and of HIV testing among MSM, which might warrant additional efforts in implementing preventative actions. Despite numerous interventions targeting the behaviour, knowledge and attitudes of MSM, an increase of STIs and HIV diagnoses have been recently observed. Outbreaks of syphilis, lymphogranuloma venereum (LGV), hepatitis C viral infection (HCV) and other STIs have been reported in multiple European cities, possibility as a result of risky sexual behaviour and extensive sexual networking [26], but also may be due to an increase in active offering of HIV and STI’s testing over the years.

A higher treatment coverage and higher percentage of HIV-positive MSM with undetectable viral load was attained in some Western European cities (Brussels, Hamburg, Brighton and Verona), indicating that when the service provision is proactive and the treatment widely available, the link between testing and treating can effectively influence the HIV epidemic.

### Generalisability and limitations

To our knowledge, this is the first paper presenting weighted estimates produced as a result of a standardised collection of GAM indicators for MSM in a large number of European cities adopting a common SGSS approach. The use of this approach (SGSS and GAM Indicators) and the active participation of key LGBT community stakeholders in the project's design and implementation, represent an asset that provides potentially usable data for both public health authorities and NGOs in each partner country.

The use of TLS and RDS methodologies within the context of a bio-behavioural survey using a participatory approach have allowed hidden and different sub-groups of MSM to be reached; groups that are usually more difficult to access through surveillance studies [28]. Nevertheless, there are some limitations that should be taken into account when interpreting the results.

First, data can only be generalised to the particular MSM attending the gay venues in each study site (in the case of TLS survey) and only to those MSM socially linked to the gay community for each specific site (in case of the RDS survey). It has also been shown in other studies that these two sampling methods can result in different sample characteristics and the differences may persist even after applying weighting corrections [28].

Second, the generalisability of the findings may also be limited by contextual factors not measured in this survey (e.g. legislation and social norms) and an ecological fallacy cannot be excluded.

Third, an additional source of bias limiting generalisability relates to the self-reported behavioural data. This is of course an issue common to all surveys covering the self-reporting of sensitive information. However, the anonymity of the data collected and the self-administration of the questionnaire, with the careful design of the questionnaire items developed and validated in all cities with the involvement of the LGBT community, may well have reduced any social desirability effect [29]. It is also worth noting that no difficulties or limitations were reported by either respondents or data collectors with regards to the use of the GAM indicators (both TLS and RDS) in terms of interpretation and utility of the items and related indicators.

Fourth, the percentage of participants who reported ever having injected drugs ranged from 1.2% in Bratislava to 19.3% in Bucharest. In the latter, estimates might be difficult to be generalised due to this sub-population of injecting drug users (IDU)-MSM and to the fact that the target sample was not reached.

Fifth, the precision of the estimates were in some cases not optimal, particularly for the group of younger MSM, due to relatively small sample sizes (< 50) and the potential for sampling bias in some cities.

Finally, an isolated limitation to the validity of the survey relates to Sofia (Bulgaria) where, due to an incorrect translation in the items questionnaire related to GAM 1.13, the indicator could not be estimated.

### Conclusions

The Sialon II project and the data generated through its implementation represent a collaborative and scientifically robust contribution to the monitoring and evaluation of the HIV epidemic across Europe, integrating the use of GAM indicators within a SGSS approach with active community involvement. The data collected provide new evidence for appropriate planning of HIV prevention campaigns among MSM, clearly responding to the urgent need of concerted use of common indicators, with a particular focus on most at risk populations such as MSM [9]. The project has actively contributed to: (i) common procedures piloting (including most-advanced sampling methods, standards research algorithms, advanced laboratory diagnostics); (ii) harmonised data collection, involving experts from different institutions and with different backgrounds; and (iii) GAM country reporting, with specific reference to MSM.
